# A Social Media Website (Supporting Our Valued Adolescents) to Support Treatment Uptake for Adolescents With Depression and/or Anxiety and Their Parents: Protocol for a Pilot Randomized Controlled Trial

**DOI:** 10.2196/12117

**Published:** 2019-01-23

**Authors:** Ana Radovic, Yaming Li, Douglas Landsittel, Bradley D Stein, Elizabeth Miller

**Affiliations:** 1 Division of Adolescent and Young Adult Medicine Department of Pediatrics University of Pittsburgh School of Medicine Pittsburgh, PA United States; 2 Department of Biomedical Informatics University of Pittsburgh Pittsburgh, PA United States; 3 RAND Corporation Pittsburgh, PA United States

**Keywords:** adolescent, adolescent health services, anxiety, depression, technology

## Abstract

**Background:**

Few adolescents who experience depression or anxiety connect to mental health treatment. Supporting Our Valued Adolescents (SOVA) is a stakeholder-informed technology intervention that consists of 2 blog-format websites—one for adolescents and another for parents. SOVA is designed to intervene on targets, which may increase the mental health treatment uptake when adolescents with depression or anxiety are identified in primary care settings.

**Objective:**

This study aims to describe the protocol for a pilot randomized controlled trial designed to refine recruitment and retention strategies, document intervention fidelity and implementation outcomes, and assess changes in health beliefs and knowledge, emotional or informational support, and parent-adolescent communication quality in adolescents and their parents.

**Methods:**

Adolescents identified with symptoms of depression or anxiety, for which a health care provider recommends treatment, and their parents will be recruited from clinics where adolescents are seen for primary care. Adolescent-parent dyads will be randomized at 1:1 to both receive the SOVA websites and enhanced usual care or enhanced usual care alone. Baseline measures and 6-week and 3-month outcomes will be collected by Web-based self-report surveys and electronic health record review. The main pilot outcome is the 6-week study retention rate. Analyses will also assess changes in health beliefs and knowledge, emotional support, and parent-adolescent communication in both adolescents and their parents.

**Results:**

The project was funded in 2017. Recruitment commenced in April 2018 and enrollment is ongoing, with completion anticipated at the end of 2019 with subsequent plans for data analysis and publication submission in early 2020.

**Conclusions:**

The findings of this research will inform the design of a multisite hybrid effectiveness-implementation randomized controlled trial examining the effectiveness and optimal implementation strategies for using SOVA in community primary care settings.

**Trial Registration:**

ClinicalTrials.gov NCT03318666; https://clinicaltrials.gov/ct2/show/NCT03318666

**International Registered Report Identifier (IRRID):**

PRR1-10.2196/12117

## Introduction

Adolescents are experiencing depression and anxiety in growing numbers, but few connect to mental health care. Almost 12% of adolescents have major depression or dysthymia and up to a third have an anxiety disorder [1]. A third of depressed adolescents experience suicidality, and 11% attempt suicide [2], resulting in US $12 billion in hospital costs [3]. Yet, only one-third of depressed adolescents receive treatment [4], and initial treatment delays average 10 years [5]. Unmet treatment needs are even more concerning for anxiety, with less than one-fifth of adolescents using mental health services [6]. Although research shows positive effects of antidepressants and cognitive behavioral therapy [7], these treatments are underused in adolescence [8,9], contributing to higher health care utilization as adults [10].

One approach to increasing access and use of mental health treatment is implementing integrated behavioral health models. These models may increase the number of adolescents receiving treatment by actively engaging patients, enabling consultation with and access to mental health professionals within primary care settings, and increasing evaluation and management by primary care providers (PCPs) [11]. By standardizing evaluation through routine screening, providing access to services, and nurturing active engagement in care [12], these models can improve adolescent depression treatment outcomes in primary care [12], where one-third of child mental health is managed [13]. However, these in-person patient engagement techniques may fail to be implemented owing to being resource intense and requiring trained professionals and practice-level changes. More commonly, depression screening is attempted without an approach to increase engagement resulting in low treatment initiation [8,14,15]. One explanation is adolescents identified through routine screening, and their parents may not be seeking mental health services [16,17], leading to a mismatch between evaluated (ie, screening results and PCP evaluation) and perceived need (ie, parent and adolescents’ views on whether they need services).

Models of mental health service use can be used to examine factors contributing to the underuse of mental health treatment [17]. The Andersen behavioral model explains how (1) predisposing characteristics (eg, age, gender, race, health beliefs, and knowledge); (2) enabling resources (eg, health insurance, income, and emotional support); and (3) need for services (both evaluated and perceived) can predict service use [18,19]. Parents’ and adolescents’ perceptions of the adolescents’ need for mental health services are known predictors of service use [16,20-23] but difficult for PCPs to address [24].

Negative health beliefs about biological explanations for mental illness and lack of confidence in treatment lead to a decreased perceived need for treatment in young adults [25] and parents [26]. A systematic review found the most important barriers in young people are lack of mental health knowledge [27] and negative beliefs about treatment [28]. Independent of access to care or cost, negative health beliefs strongly correlate with the persistent unmet need of adults who developed mental health problems in childhood [23,29].

Emotional support increases mental health services use [18,28,30] and may be important for both adolescents and their parents. Peers contribute to mental health interventions by providing hope through self-disclosure, role modeling positive behavior, and using empathy and acceptance [31]. Peer social support is influential in adolescent decision making [32]. For both adolescents and parents, social support can improve treatment expectations [33,34] and increase antidepressant acceptance [35]. By comparing their child’s emotional problems with other children, parents consider whether symptoms are developmentally normal [36]. Parents who are peer advocates help other families by sharing knowledge and addressing attitudes toward treatment [37].

Parents’ role in facilitating an adolescent to engage with mental health services is gravely important [16,20]. Impaired parent-adolescent communication may decrease help seeking for mental health problems [38,39]. Adolescents’ developmental goals of establishing autonomy in decision making and becoming independent can make communication around mental health difficult [40], especially for internalized disorders, like depression, for which adolescents may not express their symptoms aside from displaying irritability—and this can be interpreted as normalized adolescent defiance or “teen angst.” Externalizing disorders with behavioral symptoms that parents can readily observe, such as in attention deficit hyperactivity disorder, are more likely to lead to the parent requesting treatment for their adolescent [41]. Few studies that have examined communication around mental illness show evidence for silence and stigma [42], with parents feeling least comfortable discussing suicidal thoughts [43].

**Figure 1:**
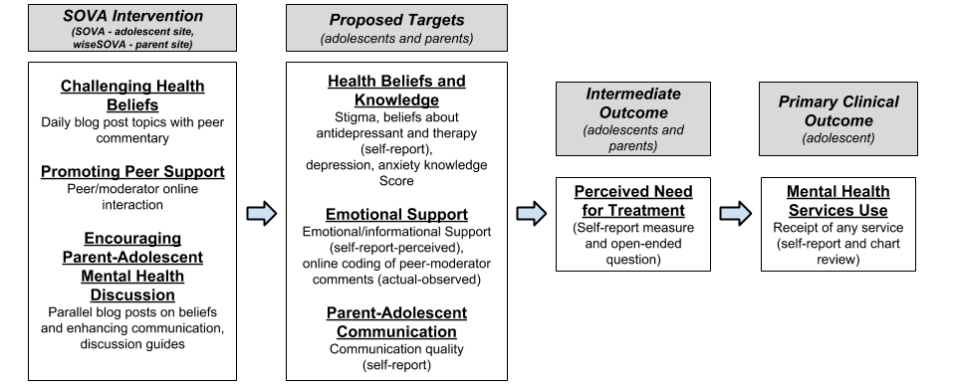
Conceptual model. SOVA: Supporting Our Valued Adolescents.

The conceptual model in Figure 1 displays how the abovementioned factors may be related. The Supporting Our Valued Adolescents (SOVA) intervention, described below, was designed to address these proposed targets by challenging health beliefs, promoting peer support, and encouraging parent-adolescent mental health discussion. Further details of the design and development of this intervention [44] and its usability testing [45] are available elsewhere.

This protocol aims to use a pilot randomized controlled trial (RCT) of SOVA compared with enhanced usual care (EUC) provided to adolescents with symptoms of depression or anxiety being referred to treatment to refine recruitment and retention strategies, document intervention fidelity and other implementation outcomes, and assess changes in health beliefs and knowledge, emotional or informational support, parent-adolescent communication quality, and explore whether there are differences in mental health service use, the proposed future main outcome. The main pilot outcome is the retention rate, with other feasibility metrics also being described.

## Methods

### Study Overview

We will conduct a 6-week pilot, single-blind RCT of SOVA in parent-adolescent dyads identified by their clinician in the course of routine medical care with symptoms of depression or anxiety and referred for a new treatment episode (no treatment in the past 3 months defined as having filled and begun taking an antidepressant and seeing a mental health therapist for at least 3 sessions). This will be a parallel treatment arm study with 1:1 allocation comparing SOVA and EUC to EUC alone. We will examine the feasibility of recruitment and retention strategies, intervention implementation, acceptability by providers, measures appropriateness, rates of missing data, and adequacy of the human subjects’ plan. In addition, we will test randomization procedures and measure mental health service use after 3 months. We will also describe changes in target mechanisms (Figure 1) as an embedded proof-of-concept study. For this study, by “retention,” we mean assessing whether recruited participants are retained in the study (as opposed to retained in treatment) and complete 6-week outcome measures as estimating the loss to follow-up will help us to plan the sample size needed for a larger effectiveness trial.

### Participants and Setting

The University of Pittsburgh Medical Center Children’s Hospital of Pittsburgh Center for Adolescent and Young Adult Health (CAYAH) has 4 clinical sites (2 urban academic and 2 suburban community) staffed by 17 adolescent health care providers (AHCPs), including pediatricians specializing in adolescent medicine, nurse practitioners, physician assistants, and pediatric/adolescent gynecologists. AHCPs provide primary care and adolescent consultative services (eg, reproductive health, chronic disease management, and mental health concerns) for young people aged up to 26 years. Patients are routinely screened for depressive symptoms with PHQ-2 [46]; if positive, AHCPs use more extensive screening tools for depression (Patient Health Questionnaire, PHQ-9) [47,48] and anxiety (Generalized Anxiety Disorder, GAD-7) [49] and a brief clinical interview to aid with diagnosis. With an integrated behavioral health care model, licensed social workers and psychologists are available to provide therapy in clinic and work in an integrated fashion with social workers who provide brief counseling and care management. Owing to the availability of on-site mental health services and the focus of the SOVA intervention on addressing attitudinal and not access barriers, this setting was chosen for this initial pilot study. To enhance recruitment, an additional affiliated academic pediatric primary care clinic, which also has access to therapists and conducts routine screening for depression, will also be included. Throughout this manuscript, AHCP will also refer to these affiliated pediatric PCPs. Some individuals who initially accept a PCP referral do not follow through with the referral [15], and a dyad who initially refuses may eventually accept treatment. Hence, recruitment will not distinguish between treatment refusers and engagers, and dyads will be recruited based on the provider determination for the need for treatment.

### Recruitment

The research team (RT) will notify AHCPs about the study through an announcement during a regularly scheduled faculty and staff meeting. Patients and parents seeking clinical services are routinely informed that they may be approached about research studies during their visit. In the waiting room and clinic rooms, recruitment posters will be visible, and a recruitment postcard will be available for patients and parents to enter their information and indicate interest in the study, as well as coloring bookmarks and colored pencils. AHCPs will receive the recruitment postcard from their patient, or they may be reminded about the study by a clinical social worker or an RT member. The back of the recruitment postcard will remind the AHCP (1) about the study inclusion criteria, that is “If you are referring this patient for depression or anxiety treatment of any kind (even a follow-up with you) and if they are between the ages of 12-19, they may qualify for the Stress and Worry study;” (2) to include a Stress and Worry patient education information in the electronic depart summary, that is a meaningful use requirement of the electronic health record (EHR) for the CAYAH clinical team; and (3) to ask the patient and parent if they want to stay after the visit to be screened. If they do want to stay to assess eligibility for the study, the AHCP can indicate the research assistant (RA) to come to the patient room, and if they do not want to stay, they can enter the research postcard in a lockbox, and the patient and parent will be contacted at a later time. Recruitment goals are to recruit about 12 adolescents per month based on the clinic volume and projected number of adolescents who should be offered treatment from other clinical studies on adolescent depression.

### Study Procedure

An RT member will use the EHR to prescreen patients, which will involve examining the EHR to confirm the inclusion or exclusion criteria that can be retrieved (see Textbox 1 for full inclusion or exclusion criteria). If criteria are met, then the RT member will proceed to contact interested patients or their parent. If in the clinic, the RT member will discuss this inclusion criteria with the referring AHCP prior to interacting with the patient and parent. Adolescents only up to 19 years are included, as the goal of the SOVA intervention is to encourage earlier initiation of treatment for depression or anxiety.

A waiver of parental permission was obtained from the Institutional Review Board for screening and study enrollment; this was asked for as the target population of the intervention includes adolescents who may have poor communication with their parent and, therefore, do not want to disclose symptoms of depression or anxiety, which would need to occur during the consent process during explanation of study purpose. For adolescents who wish to enroll in the study without their parent, all the same procedures below will be followed for the adolescent alone.

The RT member will then contact the patient and their parent if enrolled (if the RT member is available in the clinic, this will be done in-person, if not, it will be carried out over the phone). RT members will ask the adolescent screening questions from PHQ-9 and GAD-7 or obtain this information from the EHR. If an adolescent scores ≥5 on one or both PHQ-9 and GAD-7, consistent with mild symptoms, then the RT member will ask further inclusion and exclusion criteria. All study data will be collected in the Research Electronic Data Capture (REDCap), a secure Web application used to build and manage secure databases and includes capabilities, such as Web-based surveys, with branching logic and automatic scheduling, as well as randomization modules [50]. If an adolescent scores in the severe category (15-21) on the GAD-7 or severe (20-27) on the PHQ-9, the RT will contact the AHCP to assure that clinical care is in place.

**Figure 2:**
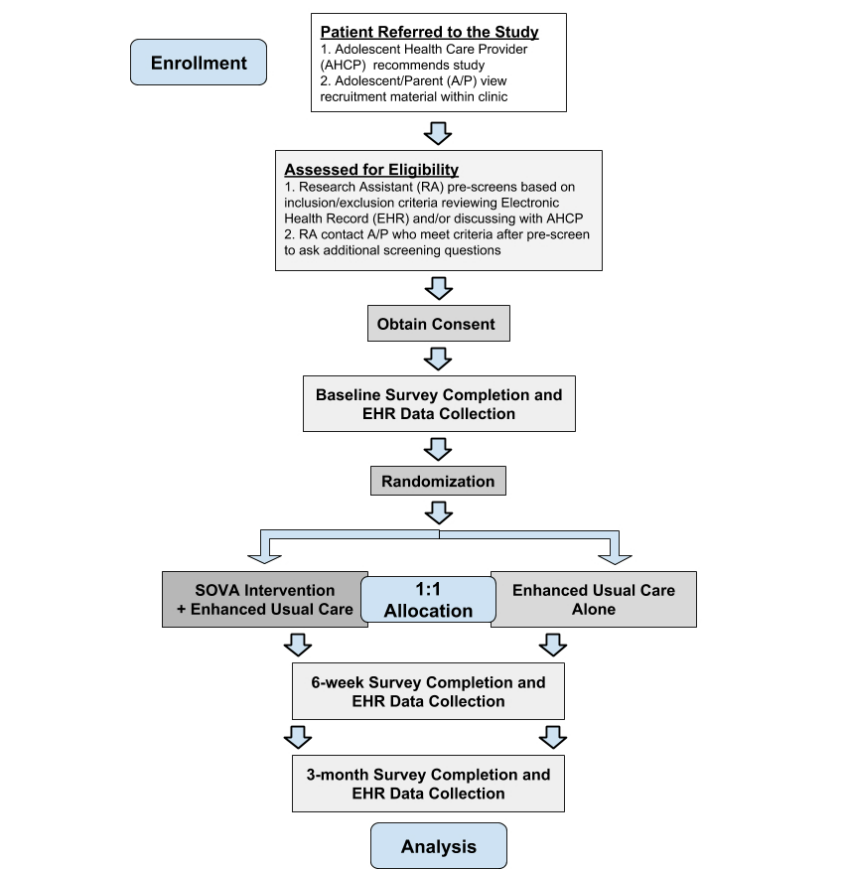
Randomized controlled trial study design. SOVA: Supporting Our Valued Adolescents.

If adolescents wish to enroll with their parent, the RT member will then obtain parental consent, and permission from the parent for the adolescent to participate in the study and assent from the adolescent (or consent from an adolescent aged ≥18 years) documented in REDCap. If adolescents wish to enroll without their parent, the RT member will obtain only adolescent assent. The RT member will notify participants regarding information for compensation, and the RT will ask them to provide a username they would like to use if they are randomized to SOVA. Then, participants will receive a baseline survey emailed from REDCap. Once the adolescent completes the baseline survey, the Principal Investigator or main RA will use the REDCap software to conduct permuted block randomization stratified by patient gender (male vs other owing to a lower number of males attending CAYAH) to randomize to one arm of the study SOVA+EUC or EUC alone. For each adolescent, if their parent is also enrolled, the parent will be assigned to the study arm the adolescent was randomized to. The randomization scheme has been generated by the statistician YL and will not be viewed by the rest of the RT. The AHCP and the RT member conducting 6-week and 3-month EHR data collection will be blinded to the study arm. Figure 2 shows a schema of the RCT design.

#### Control Arm

All study participants will receive EUC. EUC will include routine follow-up by one of the clinic social workers as is standard practice at CAYAH. The social worker tracks the treatment adherence (therapy attendance) and assists AHCPs with medication monitoring. The social worker offers to assist patients with an appointment, a process shown to increase the first appointment show rate but not increase the uptake rate (ie, the number of patients who schedule out of those referred) [51], but if patients are no longer interested in services, or multiple unsuccessful phone attempts are made (a frequent scenario), then the social worker communicates this with the referring medical provider.

As there is variability in the number of routine follow-ups each patient receives from the clinic social worker due to limited time or availability as well as due to enrolling from a pediatric primary care clinic with a slightly different procedure of following up with patients, we will further standardize EUC. In addition, each patient will receive an informational email from the secure research study email and a phone call from a team Moderator. Moderators are RT members who are volunteer graduate students with a background in social work or psychology who are supervised by the principal investigator (AR). They check for emails to the study account (emails are sent whenever there is new website activity), at least, every 3 hours. The informational email and phone call will be to convey the treatment that the AHCP recommended and ask the adolescent or parent if they are enrolled, if they have questions regarding this treatment, as well as how to contact their clinical team and crisis resources. If the adolescent or parent have questions, the Moderator will securely message the AHCP and CAYAH social worker the question and ask them to contact the adolescent or parent.

#### Intervention Arm

In addition to EUC, those participants (adolescents and parents) randomized to SOVA will receive a Web-based intervention. The websites for adolescents [52] and parents [53] are 2 separate websites where some content is public (the text of blog posts), but all social content (commenting on blog posts, creating a user profile) requires a username and log-in. All users will receive a separate username and password and adolescents will only log on to the adolescent site, and parents will only log on to the parent site. Every day, there is a new blog post published on the site. While some are written by the RT, others are written by SOVA Peer Ambassadors. These Peer Ambassadors are 14-26-year-old young people recruited to a separate study to understand the effects of blogging on psychological outcomes and resilience. Peer Ambassadors have a history of depression or anxiety and are willing to anonymously share their personal experiences on the SOVA website. All content written by Ambassadors is screened, scheduled, and edited if need be by the RT. Peer Ambassadors are also encouraged to regularly comment on others’ blog posts.

**Inclusion and exclusion criteria.**

**Inclusion criteria**

- Adolescents
  - Aged 12-19 years
  - Adolescent health care provider (AHCP) identifies depressive and/or anxiety symptoms
  - Scores at least 5 or greater on the Patient Health Questionnaire (depression) and/or Generalized Anxiety Disorder 7-item scale (anxiety) consistent with at least mild symptoms
  - AHCP recommends adolescent to initiate a new treatment episode (no treatment in the past 3 months)
  - Can read and write in English
  - Has completed the 6th grade
  - Assent (<18 years of age) or consent to study (18 or 19 years of age)

- Parents
  - Adolescent child meets the inclusion and exclusion criteria and agrees to enroll in the study
  - Can read and write in English
  - Has completed the 6th grade
  - Consents to study

- ACHPs
  - Health care provider (physician, nurse practitioner, and physician assistant) providing clinical services
  - Consents to study

**Exclusion criteria**

- Adolescents
  - Actively suicidal requiring crisis or hospitalization defined as currently having suicidal thoughts and a plan and AHCP recommends immediate crisis services or evaluation for hospitalization
  - History of receiving a psychiatric medication and/or psychotherapy at least 3 times within 2 months for depression and/or anxiety in the past 3 months
  - No access to the internet
  - No active email account

- Parents
  - No access to the internet
  - No active email account

- ACHPs
  - None

**Figure 3:**
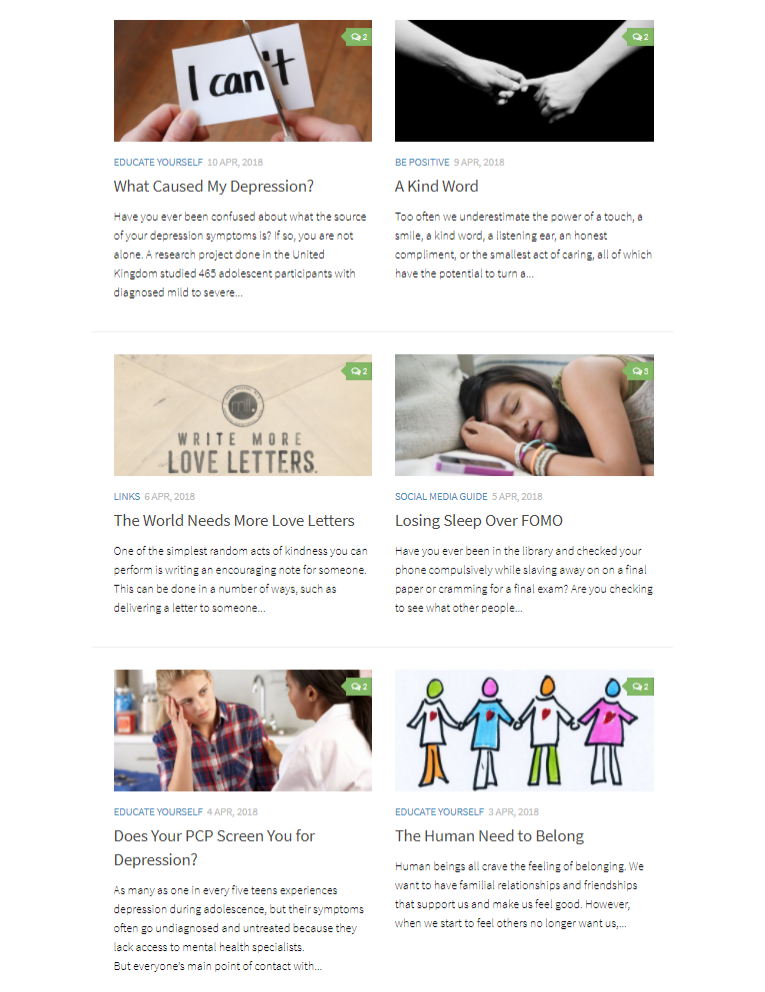
Examples of blog posts (source: SOVA, University of Pittsburgh).

Each day of the work week is designated a blog topic theme—positive posts such as uplifting quotes or stories (Monday, eg, “A Kind Word”—a post about how it feels to hear something kind from someone else), psychoeducational posts that also address negative health beliefs (Tuesday, Wednesday, eg, “How to Discuss Hard Topics with Parents” and “When I Grow Up, I Don’t Want to Be Like You”—an article discussing the impact of family mental health on whether you plan to seek help for your own), social media education posts (Thursday, eg, “Losing Sleep over FOMO”), and posts which describe other existing resources (Friday, eg, “Circle of 6”—a safety mobile app). Figure 3 presents an example of blog posts. The content on the adolescent website has a corresponding article of the same topic but modified for a parent audience on the parent website on the same day. This is to promote parent-adolescent conversation about the same type of content. Even Peer Ambassador posts are also published by the RT on the parent website so that parents may have insight into young people’s perspectives.

Every week, each participant receives an email update with blurbs of the previous week’s new posts. Currently, apps are available for Android devices of both websites that parents and adolescents can download. iPhone apps are in progress and may become available during the trial. Those who open the website regularly on the same computer or download the mobile app will also receive daily notifications of new blog post topics. On the adolescent site, users can only make comments under blog posts. If someone replies to their comment they can be notified. On the parent website, in addition to commenting, there is a discussion board feature as well, and again users are notified if there are replies to their comments on blog posts or the discussion board.

During the Moderator phone call, in addition to the information provided as part of EUC, the Moderator will ask about whether adolescents or parents have tried out and any problems experienced using the websites, explain the purpose of and content available on the websites, the role of SOVA Peer Ambassadors, encourage participation, and explain the role of the Moderator, the ground rules or community guide, and reiterate that adolescent and parent sites are separate.

For adolescents who enroll separately without their parent, we will still provide them with information to share about the wiseSOVA websites that they may provide to their parent; for EUC, adolescents will receive a letter they can provide to their parent listing the information described in the email. In this way, we will encourage parent-adolescent communication without artificially requiring it through the study consent process.

### Measures

#### Pilot Study Main Outcome: Study Retention

The study retention rate, the proportion of adolescents who access the 6-week survey in proportion to those who access the baseline survey, will be the main pilot outcome. This pilot outcome will help for planning recruitment and retention goals for a larger future RCT.

#### Implementation Outcomes

We will also examine a crude implementation outcome to understand the AHCP fidelity to the intervention by measuring the overall number of adolescents receiving information about “Stress and Worry” in their depart summary and, if possible, compare with the overall International Classification of Diseases, 10th Revision, codes of depression or anxiety made for patients seeing providers at CAYAH. This templated “depart summary” is already a part of AHCPs’ workflow as a quality measure [54] with 85% completion on average; AHCP’s will be informed about the “Stress and Worry” patient education information, which AHCP’s can elect to insert into their depart summaries. This patient education information has psychoeducation materials [55-57], a place to enter treatment recommendations, and information about the study. We will also determine the proportion of adolescents who show interest in the study compared with the number who receive “Stress and Worry” information in the depart summary. We will conduct posttrial interviews with AHCPs about the intervention acceptability.

#### Proposed Main Outcome: Mental Health Service Use

In this pilot study, multiple measures will be used to determine mental health service use, to determine which measure(s) to choose for a larger future trial. For this, we will conduct an EHR review to determine whether the medication was prescribed or filled and whether a therapist or PCP follow-up appointment(s) was attended. As some information may not be available in the EHR available to the hospital system—for example, a therapist who does not belong to the hospital system may have been seen—we will also use parent and adolescent self-report to determine services accessed by simply asking, “Have you (your child) received any treatment for depression or anxiety since the start of this study (this could include starting a new medication, seeing a professional to talk to, or follow up with your adolescent healthcare provider to talk about depression or anxiety)?” and, if yes, what treatment was received. This will be combined into one measure and mental health services accessed (yes or no) will be determined based on a positive response by either parent or adolescent, or as indicated by the EHR [14].

As multiple types of providers may be accessed when seeking help, we will use the General Help-Seeking Questionnaire [58] at the baseline to determine intention to seek help and the Actual Help-Seeking Questionnaire [59] at follow-up and after 3-months to determine what help was sought from whom.

Table 1 provides an overview of the measures, and Multimedia Appendix 1 provides a detailed description of the study measures. The proposed target mechanisms—health beliefs and knowledge about depression or anxiety, as well as peer emotional and informational support—will be measured in both adolescents and their parents. Furthermore, health beliefs will be elicited through measuring stigma and beliefs about antidepressants and therapy.

### Planned Analysis

The main summary statistic sample size is based on the retention rate; a 95% CI will be calculated. Descriptive statistics (the percentage for proportions and means and SDs for continuous measures) will be presented for all feasibility (retention and site usage) and implementation measures. Outcome measures (listed in Multimedia Appendix 1) will be summarized by the study arm and evaluated for change (from the baseline to 6 weeks) in proposed target mechanisms using the chi-square test (for dichotomous outcomes) and 2-sample *t* tests (for continuous measures) separately for adolescents and parents. The number and percentage missing will be reported for each survey item. In addition, study arms will be assessed for balance across all covariates. Content analysis will be used to code Web-based peer-peer or peer-moderator interactions for social support (emotional, empathy, positive emotional self-disclosure, and expressions of concern) [67]. Furthermore, survey data regarding communication and relationship quality will be used to explore temporal associations between parents’ and adolescents’ change scores in communication quality by the arm using correlational analyses.

At CAYAH, 3845 unique patients aged 12-19 years were seen in 2017. If up to 29.9% (1153/3845) may be identified with symptoms of depression or anxiety [1,74] and 66.9% (2576/3845) may have not had treatment in the past 3 months [4], 20.0% (772/3845) would be eligible for the study over 1 year.

The main outcome is the study retention rate. We predict it will be at least 90%; with an estimated sample size of 150 adolescents (75 per arm), the 95% CI will be 85.2%-94.8%. This would be feasible as only one-fifth of eligible patients would need to enroll. We selected this sample size as the target for this pilot because this number may also facilitate meaningful Web-based peer interaction (ie, having sufficient users online at one time). This target sample size may be reduced based on the study timeline and actual recruitment rates, as this number would not be required for determining pilot outcomes.

**Table 1:** Measures to be obtained and proposed target mechanisms adolescents and parents.

Construct and method of operationalization		Measure	Timepoint		
			Baseline	6 weeks	3 months
**Health beliefs**					
	1. Stigma	1. Depression Stigma Scale [60]	1, 2, 3	1, 2, 3	N/A^a^
	2. Beliefs about antidepressants	2. Resistance to Antidepressant Use Questionnaire, Antidepressant Meanings Scale [61]	1, 2, 3	1, 2, 3	N/A
	3. Beliefs about therapy	3a. Adolescent: Barriers to Adolescents Seeking Help [62,63]; 3b. Parent: Parental Barriers to Help Seeking Scale [64]	1, 2, 3	1, 2, 3	N/A
**Mental health knowledge**					
	1. Depression knowledge	1. Depression Literacy Questionnaire [65]	1, 2	1, 2	N/A
	2. Anxiety knowledge	2. Anxiety Literacy Questionnaire [65]	1, 2	1, 2	N/A
**Peer emotional /informational support**					
	1. Perceived emotional/informational support	1. Emotional/Informational Subscale from the Medical Outcomes Study Social Support Survey [66]	1	1, 2	N/A
	2. Actual/observed emotional/informational support	2. Online coding of peer and moderator comments for types of social support [67]	1	1, 2	N/A
**Communication quality**					
	1. Parent-adolescent communication quality	1. Parent-Adolescent Communication Scale [68]	1	1	N/A
**Perceived need for treatment**					
	1. Perceived need for treatment	1a. Open-ended question about whether adolescent/child needs any mental health service [21]; 1b. the General-Practice Users Perceived-Need Inventory [69]	1a, 1b	1a, 1b	N/A
**Proposed main outcome: mental health service use**					
	1. Intention to seek services	1. General Help Seeking Questionnaire [58]			
	2. Receipt of any mental health treatment	2a. Combined measure using Electronic Health Record Chart Review and parent/adolescent self-report [14]; 2b. Actual Help Seeking Questionnaire [58,59]	1	2a, 2b	N/A
**Exploratory clinical outcomes**					
	1. Depressive Symptoms	1. Adolescent: Depressive Symptoms: Patient Health Questionnaire-9 item [47]	1, 2, 3, 5	1, 2, 3, 4a, 4b, 5	4a, 4b
	2. Anxiety Symptoms	2. Adolescent: Anxiety Symptoms: Generalized Anxiety Disorder Scale-7 item [49]	1, 2, 3, 5	1, 2, 3, 4a, 4b, 5	4a, 4b
	3. Functioning	3a. Adolescent: Multidimensional Adolescent Functioning Scale [70]; 3b. Parent: Columbia Impairment Scale-Parent [71]	1, 2, 3, 5	1, 2, 3, 4a, 4b, 5	4a, 4b
	4. Continued mental health service use	4a. Combined measure using Electronic Health Record Chart Review and parent/adolescent self-report [14]; 4b. Actual Help Seeking Questionnaire [58,59]	1, 2, 3, 5	1, 2, 3, 4a, 4b, 5	4a, 4b
	5. Relationship quality	5. Parent-child connectedness [72]			
**Descriptive covariates**					
	1. Demographics	1. Age, gender, sexuality (adolescent only), race, ethnicity, education, health insurance (parent only – asked of child), transportation, socioeconomic status	1, 2, 3, 4a, 4b	4b	N/A
	2. Treatment history	2. History of medication/therapy in adolescent [73]	1, 2, 3, 4a, 4b	4b	N/A
	3. Treatment provider recommends	3. Chart review for treatment recommendation	1, 2, 3, 4a, 4b	4b	N/A
	4a. Parental mental health history; 4b. Parental receipt of mental health treatment	4a. Parent: Parental self-report mental health history [14]; 4b. Parent: Actual Help Seeking Questionnaire [58,59]	1, 2, 3, 4a, 4b	4b	N/A

^a^N/A: not applicable.

## Results

We expect to determine (1) the feasibility of retaining 90% of individuals for follow-up; (2) implementation outcomes estimating the intervention fidelity; (3) intervention engagement; (4) provider acceptability; (5) appropriateness of measures, amount of missingness; (6) adequacy of human subjects’ plan; (7) change in target mechanisms; and (8) extent of Web-based social support achieved, measured by coded Web-based communications.

The University of Pittsburgh Human Research Protection Office provided ethics approval for this study (PRO17070601). It has been registered with ClinicalTrials.gov Protocol Registration and Results System (#NCT03318666).

The project was funded in 2017. Recruitment commenced in April 2018 and enrollment is ongoing, with completion anticipated at the end of 2019 with subsequent plans for data analysis and publication submission in early 2020.

## Discussion

This paper describes the protocol for a pilot RCT of the SOVA intervention to refine recruitment and retention strategies, document intervention fidelity and implementation outcomes, and assess changes in health beliefs and knowledge, emotional or informational support, parent-adolescent communication quality, and perceived need for treatment. The findings of this research will inform the study design of a larger multisite hybrid effectiveness-implementation RCT examining the effectiveness of and optimal implementation strategies for using SOVA in community primary care settings. The main outcome measured in this larger trial will be whether SOVA increases the mental health service use compared with usual care. This research will help inform strategies to increase service use of evidence-based depression and anxiety treatments in adolescents and provide PCPs with a tool to address adolescent and parent concerns and attempt to increase their perceived need for treatment.

We considered several iterations of this study design with regards to setting and timing of the intervention. This study will be conducted in a clinical setting that already has an integrated behavioral health model in place. Hence, EUC may also have a strong effect on the proposed main outcome, mental health service use. As a pilot, this study was not designed to determine the effectiveness of SOVA. The specific setting was chosen because of the higher prevalence of primary care and consultative care patients seen with depression and anxiety symptoms who are referred for treatment, as this will facilitate recruitment and is not thought to influence measuring change in the target outcomes. Concurrently, we are conducting preimplementation focus groups in community pediatric primary care settings to inform how PCPs may want to introduce SOVA in that setting. This data will inform how to measure implementation outcomes and conduct a future large hybrid effectiveness-implementation trial in community primary care settings.

Another consideration of the study design was the timing of the intervention with relation to a clinic appointment. Ideally, patients and their parent should receive the intervention immediately after the clinic appointment, but due to requirements to screen, consent, and complete baseline surveys for both the parent and adolescent prior to randomization, this timing may be delayed. Our desire was to use a more pragmatic study design that would simulate how the intervention may be introduced in routine practice, but then place less burden on clinicians seeing patients and on parents and adolescents (eg, not requiring in-person completion of study measures and consent). Another design would have been prescreening or consenting patients prior to scheduled appointments. We will gather data on timing of intervention administration to clinician visit and use this to inform the study design of the larger RCT.

These pilot data are expected to inform the planning of a hybrid effectiveness-implementation study of a technology intervention that should facilitate the more rapid translation of study results into practice.

## Appendix

Multimedia Appendix 1 Detailed description of measures.

## Abbreviations

AHCP: adolescent health care provider
CAYAH: Center for Adolescent and Young Adult Health
EHR: electronic health record
EUC: enhanced usual care
GAD-7: Generalized Anxiety Disorder Scale 7-item
PCP: primary care provider
PHQ-9: Patient Health Questionnaire 9-item
RA: research assistant
RCT: randomized controlled trial
REDCap: Research Electronic Data Capture
RT: research team
SOVA: Supporting Our Valued Adolescents
