# Supplementary material for: A Social Media Website (Supporting Our Valued Adolescents) to Support Treatment Uptake for Adolescents With Depression and/or Anxiety and Their Parents: Protocol for a Pilot Randomized Controlled Trial
Source: JMIR Res Protoc. 2019 Jan 23;8(1):e12117. doi: 10.2196/12117 (PMC6364211; doi:10.2196/12117)
Supplement: Multimedia Appendix 1 [file resprot_v8i1e12117_app1.pdf]

Multimedia Appendix: Detailed Description of Measures

Descriptive Covariates

At baseline, adolescents will be asked their age, gender, sexuality (i.e. identity, attraction, behaviors), race, ethnicity, education (i.e. last grade level completed), ease of finding transportation, and socioeconomic status (SES and community ladder from the MacArthur Subjective Social Status Scale)[1]. Parents will also be asked these questions of themselves except for sexuality. Parents and adolescents will be asked about the child's history of ever receiving mental health treatment (i.e. accessing therapy or ever being prescribed medication for a personal or emotional problem) and either the adolescent's or the parent's positive report of treatment will be counted[2] as adolescents may not recall receiving treatment as a child and parents may be unaware of adolescent's confidential mental health treatment. Parents only will be asked about whether the child has health insurance and if yes whether it is private or Medicaid/CHIP (Children's Health Insurance Program), about the parent's history of ever receiving mental health treatment themselves, and the parent's current history (past 6 weeks) of help-seeking for a personal or emotional problem using the Actual Help-Seeking Questionnaire[3] (described below). A chart review of the electronic health record will be conducted to obtain information on the current treatment recommendation by the AHCP to note what type of treatment was recommended, options including any of the following: 1) working on self-care such as sleep, 2) starting a new medication for depression or anxiety, and if yes, which medication and the dose, duration, 3) seeing a professional to talk to, and if yes, which professional, and/or 4) following up with the AHCP (or PCP if AHCP is not the PCP), and if yes, within what timeframe.

Proposed Target Mechanisms

Health Beliefs and Mental Health Knowledge

Stigma will be measured using the Depression Stigma Scale,[4] an 18 item self-report scale constructed by Griffiths et al. which breaks down into two sub-scales measuring personal stigma or an individual's personal beliefs about stigma toward depression and perceived stigma or an individual's beliefs about the stigma others may have toward individuals with depression. Overall, the Depression Stigma Scale has a Cronbach's α of 0.78, the total scale score ranges from 0-36 and subscale scores range from 0-18 with higher scores indicating a higher level of stigma.

Beliefs about antidepressants will be measured through the Resistance to Antidepressant Use Questionnaire (RAUQ) and the Antidepressant Meanings Scale (AMS).[5] The RAUQ provides three short vignettes and asks likelihood/agreement on a 6-point Likert scale to (1) take antidepressants if they were depressed, (2) agree with a friend or family member's

decision to take antidepressants if they were depressed, and (3) prescribe antidepressants to depressed individuals if they were a psychiatrist or doctor. The RAUQ has a Cronbach's α of 0.78, the total score ranges from 0-18, with higher scores indicating less resistance to antidepressant use. The AMS presents a short vignette describing that you have symptoms of depression for one month and that you see your doctor who recommends an antidepressant. The scale asks to rate how worried (0-6) you would be that (1) you would become addicted to the antidepressant, (2) it would change your personality, (3) you would have difficulty stopping the antidepressant, and (4) it would have bad side-effects like fatigue, headaches, lower sex drive. The AMS has a Cronbach's α of 0.82, the total score ranges from 0-24, with higher scores indicating greater fear about undesired effects of antidepressant use.

Beliefs about therapy will be measured by the Barriers to Adolescent Help Seeking Scale (BASH) – adolescent version and parent versions. The brief version, BASH-B, will be used for this study. The BASH-B contains 11 of the original 37 questions from the longer BASH scale [6] and specifically queries those barriers to seeking professional psychological help.[7] Each of the 11 items is rated using a 6-point scale in terms of agreement with higher scores indicating greater belief-based barriers to seeking professional psychological help such as concerns about therapist confidentiality. The BASH-B has a Cronbach's α of 0.84 and the scale was found to be negatively associated with intentions to seek help and quality of prior mental health care as rated by adolescents.[7] A parental version of the BASH-B will be used to assess parents' barriers to seeking professional help for their adolescent's problems (ex. Even if I wanted to, I wouldn't have time to get professional help for my child's problem(s)). This 11-item scale is scored the same way and has been found to have a Cronbach's α of 0.80.[8]

Mental health knowledge about depression and anxiety will be assessed by the Depression Literacy Scale (D-Lit) and the Anxiety Literacy Scale (A-Lit) respectively in both parents and adolescents.[9] Both literacy questionnaires have 22 items ranked as true, false, and don't know with correct responses receiving 1 point, for a possible range of 0-22, with higher scores representing greater knowledge. The D-Lit was found to have a Cronbach's α of 0.70 and adequate test-retest reliability (r = .71); the A-Lit was found to have a Cronbach's α of 0.76 and very good test-retest reliability (r = .83).[9]

Peer Emotional and Informational Support

We will use the Emotional/Informational subscales from the Medical Outcomes Study (MOS) Social Support Survey to examine perceived emotional and informational support in adolescents and parents.[10] These subscales will be used as they are types of social support which may be garnered through interaction in an anonymous online support community (as opposed to tangible social support). These subscales have 8 questions

asking how often an individual has access to different types of social support; the range of the scale is 0-100 and the emotional/information subscale has a Cronbach's α of 0.96.[10]

We will also examine online content for evidence of social support. Individuals randomized to the website intervention's comments will be downloaded and coded for different types of social support.[11] We will describe out of all comments, how often social support is provided. This will be purely descriptive as a comparison between those randomized to SOVA and those randomized to EUC cannot be made.

Parent-Adolescent Communication Quality

We will use the Parent-Adolescent Communication Scale in both parents and adolescents to understand communication quality.[12] This scale includes a 20-item Adolescent Form and a 20-item Parent Form with subscales for degree of openness in family communication (Cronbach's α of 0.87) and extent of problems in family communication (Cronbach's α of 0.78). We exchanged the original term mother/father for parent or guardian. An example item from the problem scale is "my parent or guardian (child in parent scale) nags/bothers me" and an example item from the openness scale is "It is very easy for me to express all my true feelings to my parent or guardian (child in parent scale)." Items are answered on a 5-point Likert scale of agreement, with problem statements being reverse scored, and a higher number indicating more positive parent-adolescent communication (range 20-100). Balanced family type from the Circumplex Model has been associated with higher communication scores.[13]

Perceived Need for Treatment

The parent and adolescent will both be asked an open-ended question about their perceptions of whether the adolescent needs any mental health service, "Do you believe that you (your child) need(s) any mental health service (this includes getting help from a mental health professional like a counselor or psychologist and/or being prescribed a medication for any personal or emotional problems)?" with a yes/no response.[14] In addition, they will also be asked to respond to the General-practice Users Perceived-need Inventory (GUPI).[15] The GUPI is an instrument developed for primary care settings which asks patients if they feel they would like a general practitioner (we will use the term primary care provider or PCP) to help with emotional problems, specifically regarding information, medication, counseling, social intervention (housing), or skills training (improve ability to work, care for self, etc.). For each item, individuals are asked to respond with whether they would like help, don't need help, or already are getting help for the specific problem. At the end of the GUPI the individual is asked about several barriers to getting the help. Higher scores on the GUPI have been associated with poorer general health.[15]

Proposed Main Outcome: Mental Health Service Use

As multiple types of providers may be accessed when seeking help, we will use the General Help Seeking Questionnaire (GHSQ)[17] at baseline to determine intention to seek help and the Actual Help Seeking Questionnaire (AHSQ)[3] at follow-up and at 3-months to determine what help was sought from whom. This will also help determine, whether another individual such as a friend or a pastor, was substituted for seeing a therapist as recommended by the PCP. Both the GHSQ and AHSQ provide a list of potential individuals from whom help may be sought for a personal or emotional problem. The GHSQ asks likelihood of seeking help (Likert scale 1-7) from these individuals and the AHSQ asks whether help from the individual was sought and to describe the type of problem for which help was sought. We modified the original scale to: specify in the next 6 weeks (GHSQ) or in the past 6 weeks (AHSQ); provide an example for personal or emotional problem "(example: feeling very anxious/stressed, feeling very depressed or in a low mood); and used the following list: partner (e.g. significant boyfriend or girlfriend), friend (not related to you), parent/guardian, other relative/family member, mental health professional (counselor/social worker/psychologist/psychiatrist), help line (phone number to call/text in crisis), doctor/healthcare provider (doctor/provider you see for yearly physicals), teacher, someone else not listed above, coach, religious person (example: priest, imam, rabbi), I would not seek help from anyone. These measures have been validated in adolescent samples, and have been found to be flexible to a range of contexts. The GHSQ for personal or emotional problems has a Cronbach's α of 0.70 and test-retest reliability assessed over a three-week period was 0.86.[18]

Exploratory Clinical Outcomes

Depressive Symptoms

Depressive symptoms will be measured at baseline and 6 weeks using the Patient Health Questionnaire 9 modified for adolescents (PHQ-9). This scale inquiries about the duration (not at all, several days, more than half the days, nearly every day) of 9 different depressive symptoms experienced over the prior 2 weeks. It also asks about dysthymia experienced over the past year, and whether symptoms have made it difficult for the adolescent to function. The first 9 questions are scored on a 0-3 scale with a scale range of 0-27. This scale has been validated in adolescent samples and a score of 11 or higher has found to have an 89.5% sensitivity and 77.5% specificity when compared to an independent structured mental health interview.[23]

Anxiety Symptoms

Anxiety symptoms will be measured at baseline and 6 weeks using the Generalized Anxiety Disorder 7-item scale (GAD-7).[24] This scale also inquiries about symptoms experienced over the past 2 weeks. It asks duration of 7 symptoms with the same options as the PHQ-9 described above as well as asking one question about how difficult the symptoms have

made it to function. The first 7 questions are scored on a 0-3 scale with a scale range of 0-21. The GAD-7 has been validated in adolescent samples and a score of 11 or higher had an optimum specificity (100%) and sensitivity (97%) for detecting moderate anxiety when compared to the Clinical Global Impression-Severity score consistent with moderate illness.[25]

Functioning

The Multidimensional Adolescent Functioning Scale (MAFS) will be used at baseline and 6-weeks to assess adolescent interpretation of their functioning,[26] and the Columbia Impairment Scale – Parent version will be used to assess parent interpretation of the adolescent's functioning.[27] The MAFS asks adolescents to rate how well a set of 23 statements describe their situation on a 1-4 Likert scale, as well as one question about current letter grades. The statements assess functioning within general functioning (ex. I am pleased with how my life is going), family-related functioning (ex. My parents' rules are reasonable), and peer-related functioning (ex. My friends are often disappointed in me). The three scales have different ranges: MAFS-General functioning ranges 0-40, MAFS-Family functioning ranges 0-28, and MAFS-Peer functioning ranges 0-24. The MAFS had adequate internal consistency (0.75-0.91) and in a general adolescent sample was found to be stable across repeated measurements and genders. The Columbia Impairment Scale asks parents to rate how much of a problem on a 0-5 scale the adolescent has with 13 different items which map onto 4 areas of functioning including interpersonal relations, broad psychopathological domains, functioning in job or schoolwork, and use of leisure time. The overall score ranges from 0-52 and provides a global measure of impairment. The parental version of the scale has been found to have high internal consistency and excellent test-retest reliability, and has been validated against a clinician score on the Children's Global Assessment Scale.[27, 28]

Continued mental health service use

The SOVA intervention is directed at increasing use of mental health services, and the target mechanisms described are specifically thought to increase perceived need for services which would lead to initiation of service use. Continued use of services may indeed involve different mediators. To understand this further, we will add measuring continued service use as a secondary outcome measure at 3 months so that we can explore whether the mediators we propose are also related to this outcome. This measure will be obtained in the same way as described above under the proposed main outcome.

Relationship Quality

The Parent-Child Connectedness scale will be used to measure relationship quality with high internal consistency (Cronbach's α 0.87).[29] Both a child and parent version are

available and will be administered at baseline and at 6 weeks. This scale asks 5 questions on a 1-5 Likert scale of agreement regarding satisfaction with and closeness with the parent/child. We will adapt the scale to ask specifically about connectedness with the parent/guardian who is in the study with the child; and regarding the child who is in the study with the participating parent. The score is mean scale score, with 1 representing low connectedness and 5 representing high connectedness.

1. Adler, N., Stewart, J., and Group, P.W. *Research/psychosocial notebook*. Research Network on SES and Health 2007 [cited 2018 January 24]; Available from: http://www.macses.ucsf.edu/research/psychosocial/subjective.php.
2. Carlton, P.A. and Deane, F.P. Impact of attitudes and suicidal ideation on adolescents' intentions to seek professional psychological help. Journal of Adolescence 2000; 23: p. 35-45.
3. Rickwood, D.J. and Braithwaite, V.A. Social-psychological factors affecting help-seeking for emotional problems. Soc Sci Med 1994; 39(4): p. 563-72.
4. Griffiths, K.M., Christensen, H., Jorm, A.F., Evans, K., and Groves, C. Effect of web-based depression literacy and cognitive-behavioural therapy. Br J Psychiatry 2004; 185: p. 342-9.
5. Cohane, G., *Psychological Barriers to Antidepressant Use in Adolescents*, in *Clinical Psychology*. 2008, Clark University: Worcester, Massachusetts. p. 92.
6. Kuhl, J., Jarkon-Horlick, L., and Morrissey, R.F. Measuring barriers to help seeking behavior in adolescents. Journal of Youth and Adolescence 1997; 26: p. 637-650.
7. Wilson, C.J., Deane, F.P., and Ciarrochi, J. Can hopelessness and adolescents' beliefs and attitudes about seeking help account for help negation? J Clin Psychol 2005; 61(12): p. 1525-39.
8. Bates, L. "Predictors of Help Seeking Among Early Adolescents: Stages and Factors" (2010). Electronic Theses and Dissertations. Paper 490.
9. Gulliver, A., Griffiths, K.M., Christensen, H., Mackinnon, A., Calear, A.L., Parsons, A., Bennett, K., Batterham, P.J., and Stanimirovic, R. Internet-based interventions to promote mental health help-seeking in elite athletes: an exploratory randomized controlled trial. J Med Internet Res 2012; 14: p. e69.
10. Sherbourne, C.D. and Stewart, A.L. The MOS social support survey. Soc Sci Med 1991; 32(6): p. 705-14.
11. Wang, Y.C., Kraut, R.E., and Levine, J.M. Eliciting and receiving online support: using computer-aided content analysis to examine the dynamics of online social support. J Med Internet Res 2015; 17(4): p. e99. 4419194.
12. Barnes, H. and Olson, D., *Parent-adolescent communication scale*, in *Family Inventories: Inventories used in a national survey of families across the family life-cycle*, D. Olson, et al., Editors. 1982, Family Social Science: St. Paul. p. 33-48.
13. Barnes, H.L. and Olson, D.H. Parent-Adolescent Communication and the Circumplex Model. Child Development 1985; 56(2): p. 438-447.

14. Wu, P., Hoven, C.W., Cohen, P., Liu, X., Moore, R.E., Tiet, Q., Okezie, N., Wicks, J., and Bird, H.R. Factors associated with use of mental health services for depression by children and adolescents. Psychiatr Serv 2001; 52(2): p. 189-95.
15. McNab, C. and Meadows, G., *Brief assessment of perceived need for mental health care - development of an instrument for primary care use*, N.D. Initiative, Editor. 2004, Monash University: Australia. p. 48.
16. Breland, D.J., McCarty, C.A., Zhou, C., McCauley, E., Rockhill, C., Katon, W., and Richardson, L.P. Determinants of mental health service use among depressed adolescents. Gen Hosp Psychiatry 2013.
17. Rickwood, D., Deane, F.P., Wilson, C.J., and Ciarrochi, J.V. Young people's help-seeking for mental health problems. Australian e-Journal for the Advancement of Mental Health 2005; 4(3): p. 34.
18. Wilson, C.J., Deane, F.P., Ciarrochi, J.V., and Rickwood, D. Measuring help seeking intentions: Properties of the General Help Seeking Questionnaire. Canadian Journal of Counseling 2005; 39(1): p. 15-28.
19. Reineck, L.A. and Kahn, J.M. Quality measurement in the Affordable Care Act: a reaffirmed commitment to value in health care. Am J Respir Crit Care Med 2013; 187(10): p. 1038-9.
20. Health, N.I.o.M. *Anxiety Disorders*. [cited 2015 December 29]; Available from: http://www.nimh.nih.gov/health/topics/anxiety-disorders/index.shtml.
21. *Depression in Children and Adolescents (Fact Sheet): National Institute of Mental Health*. [cited 2015 May 21]; Available from: http://www.nimh.nih.gov/health/publications/depression-in-children-and-adolescents/index.shtml.
22. Psychiatry, A.A.o.C.a.A. *Anxiety Disorders Resource Center*. [cited 2015 December 29]; Available from: http://www.aacap.org/aacap/families_and_youth/resource_centers/Anxiety_Disorder_Resource_Center/Home.aspx.
23. Richardson, L.P., McCauley, E., Grossman, D.C., McCarty, C.A., Richards, J., Russo, J.E., Rockhill, C., and Katon, W. Evaluation of the Patient Health Questionnaire-9 Item for detecting major depression among adolescents. Pediatrics 2010; 126(6): p. 1117-23.
24. Spitzer, R.L., Kroenke, K., Williams, J.B., and Lowe, B. A brief measure for assessing generalized anxiety disorder: the GAD-7. Arch Intern Med 2006; 166(10): p. 1092-7.
25. Mossman, S.A., Luft, M.J., Schroeder, H.K., Varney, S.T., Fleck, D.E., Barzman, D.H., Gilman, R., DelBello, M.P., and Strawn, J.R. The Generalized Anxiety Disorder 7-item scale in adolescents with generalized anxiety disorder: Signal detection and validation. Ann Clin Psychiatry 2017; 29(4): p. 227-234a. PMC5765270.
26. Wardenaar, K.J., Wigman, J.T., Lin, A., Killackey, E., Collip, D., Wood, S.J., Ryan, J., Baksheev, G., Cosgrave, E., Nelson, B., and Yung, A.R. Development and validation of a new measure of everyday adolescent functioning: the multidimensional adolescent functioning scale. J Adolesc Health 2013; 52(2): p. 195-200.
27. Bird, H., Shaffer, D., and Fisher, P. The Columbia Impairment Scale (CIS): Pilot findings on a measure of global impairment for children and adolescents. International Journal of Methods in Psychiatric Research 1993; 3(3): p. 9.
28. Bird, H.R., Andrews, H., Schwab-Stone, M., Goodman, S.H., Dulcan, M., Richters, J., and Gould, M.S. Global measures of impairment for epidemiologic and clinical use with

children and adolescents. International Journal of Methods in Psychiatric Research 1996; 6(4): p. 295-307.
29. Williams, K.A. and Chapman, M.V. Unmet health and mental health need among adolescents: the roles of sexual minority status and child-parent connectedness. Am J Orthopsychiatry 2012; 82(4): p. 473-81.
